# Iliac Bone Corridors to Host the Transiliac Internal Fixator—An Experimental CT Based Analysis

**DOI:** 10.3390/jcm10071500

**Published:** 2021-04-04

**Authors:** Paul Schmitz, Maximilian Kerschbaum, Philipp Lamby, Siegmund Lang, Volker Alt, Michael Worlicek

**Affiliations:** 1 Department of Trauma Surgery, University Medical Center Regensburg, Franz-Josef-Strauss-Allee 11, 93053 Regensburg, Germany; maximili-an.kerschbaum@klinik.uni-regensburg.de (M.K.); siegmund.lang@klinik.uni-regensburg.de (S.L.); volker.alt@klinik.uni-regensburg.de (V.A.); michael.worlicek@klinik.uni-regensburg.de (M.W.); 2 Department of Plastic and Reconstructive Surgery, University Medical Center Regensburg, Caritas St. Josef, Landshuter Strasse 65, 93053 Regensburg, Germany; plamby@caritasstjosef.de

**Keywords:** pelvic ring fracture, transiliac internal fixation, TIFI, rod-screw system, minimally invasive pelvic surgery, anterior pelvic plane, APP

## Abstract

Background: The transiliac internal fixator (TIFI) is a novel minimally invasive surgical procedure to stabilize posterior pelvic ring fractures. Two bone corridors with different lengths, widths, and angulations are suitable to host screws in the posterior iliac wing. While the length and the width have been described previously, the angulation has not been determined yet. Methods: We created a computer tomography-based 3D-model of 40 patients (20 women, 20 men). The possible bone corridors to host the ilium screws for the TIFIcc (cranio-caudal) and the TIFIdv (dorso-ventral) procedure were identified. After reaching the optimal position, the angles in relation to the sagittal and axial plane were measured. The anterior pelvic plane was chosen as the reference plane. Results: The mean angle of the TIFIcc screws related to the axial plane was 63.4° (±1.8°) and to the sagittal plane was 12.3° (±1.5°). The mean angle of the TIFIdv screws related to the axial plane was 16.1° (±1.2°) and to the sagittal plane was 20.1° (±2.0°). In each group, a high constancy was apparent irrespective of the age or physical dimension of the patient, although a significant gender-dependent difference was observed”. Conclusions: Due to a high inter-individual constancy in length, width, and angulation, bone corridors in the posterior iliac wing are reliable to host screws for posterior pelvic ring fixation irrespective of each individual patient’s anatomy.

## 1. Introduction

Minimally invasive surgery is requested in all surgical disciplines. The aim of minimally invasive surgery is to reduce the local tissue damage. Reducing local tissue damage maintains tissue function as much as possible. Furthermore, the risk of surgical site infection is minimized. Finally, yet importantly, it provides a cosmetic benefit for the patient.

During the last decades in trauma surgery, approaches to the pelvis were developed from large incisions [1,2,3,4] to minimally invasive approaches [5,6,7,8]. The increasing amount of minimally invasive surgical techniques is based on the increasing knowledge of the anatomy and surgical procedures combined with the increasing amount of sophisticated implants.

**Figure 1:**
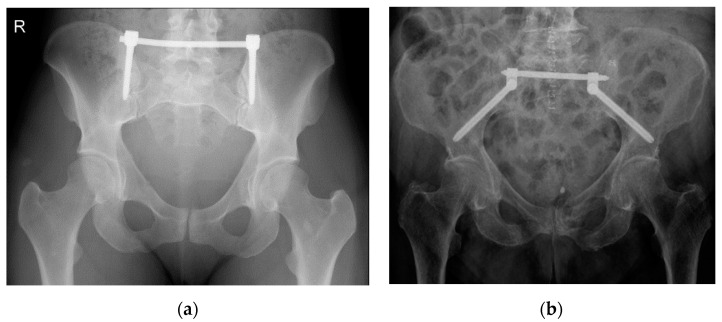
X-ray of a transiliac internal fixator (TIFI)-osteosynthesis in cranio-caudal (**a**) and dorso-ventral (**b**) orientation.

A minimally invasive procedure to stabilize an unstable posterior pelvic ring fracture is the transiliac internal fixation using an internal screw-rod system [9,10] (Figure 1). As described by Füchtmeier et al., a longitudinal incision of the skin and the fascia is performed 1 cm lateral and 2 cm above the posterior superior iliac spine. After that, the iliac crest is perforated by a pedicle finder and an ilium screw with a diameter of 7 mm and a length of up to 60 mm can be inserted in cranio-caudal direction parallel to the posterior gluteal line [9].

To account for the increasing amount of fragility fractures of the pelvis [11], Schmitz et al. modified this procedure [10] by inserting the screws at the posterior inferior iliac spine and heading towards the anterior inferior iliac spine. As a result, a screw with a length of up to 140 mm and a diameter of 8 mm can be achieved [12]. It is recommended to insert the screws under intraoperative fluoroscopic control using obturator oblique–outlet and standard lateral pelvic views [10,11,12]. After correct placement of the ilium screws, a transverse rod is inserted in a minimally invasive manner below the fascia of the spinal muscles connecting the ilium screws to each other. This way, the broken fragments of the posterior pelvic ring are merged by an angular stable implant [10]. 

The primary aim of this investigation was to measure the angles (in relation to the axial and the sagittal plane) that have to be chosen to insert the ilium screws in either of the above-mentioned TIFI procedures. Based on this experimental CT analysis, a recommendation can be given to the pelvic trauma surgeon, thereby increasing the safe screw placement of this minimally invasive procedure and reducing time consuming intraoperative fluoroscopy at the same time.

## 2. Materials and Methods

This study is based on the CT data of healthy uninjured pelves that were performed for another reason than this experimental analysis. This study was carried out in accordance with the Declaration of Helsinki and approved by the ethics committee at the University of Regensburg, Germany (Institutional Review Board Number 17-813-104). 

**Figure 2:**
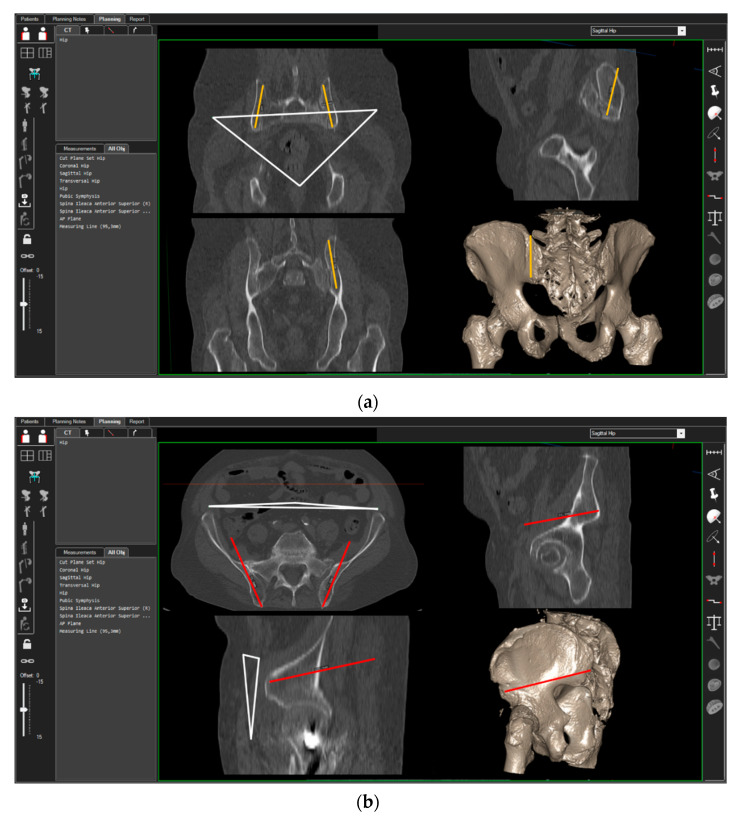
Setting of the 3D-CT-based measurement of the ilium screws in cranio-caudal (**a**) and dorso-ventral (**b**) orientation. White triangle = anterior pelvic plane (APP); orange line = position of the TIFIcc, red line = position of the TIFIdv.

3D-CT scans of 40 randomly chosen patients (20 women, 20 men) were analyzed. CT measurements were carried out using the ‘semi-automatic’ function of a digital 3D‑CT-based planning software (Modicas, Erlangen, Germany). This software offers the possibility to assess the pelvis in three dimensions, to exactly determine the axes, and to automatically calculate angles and measure distances (Figure 2). First, the pelvis was virtually aligned in order to bring the anterior pelvic plane (APP) in congruence with the coronal plane in order to have a constant starting point. The APP is defined as the triangle between the pelvic symphysis and both anterior superior iliac spines (ASIS) [13] (Figure 2, please see the white triangle). In this context, fiducial landmarks from the frontal, sagittal, and axial view were identified and the specimens’ preoperative and postoperative position was carefully adjusted in order to exclude any rotational errors during the CT measurements.

The possible entry points for the iliac screws in the posterior iliac crest were identified. For the TIFI placed in cranio-caudal direction (TIFIcc), the entry point was chosen 1 cm lateral and 2 cm above the posterior superior iliac spine. The screw (Screw-TIFIcc) was oriented in the direction of the ischiadic notch parallel to the posterior gluteal line, as described previously [9]. For the alternative stabilization technique described by Schmitz et al. [10], the ileum screw was placed in a dorso-ventral direction (TIFIdv) and the entry point was chosen 1 cm above the posterior inferior iliac spine (PIIS). The screw (Screw-TIFIdv) was positioned in the direction of the anterior inferior iliac spine (AIIS).

The next step was to ensure that the screws were positioned in a way that they were surrounded by cancellous bone and that no contact to cortical bone existed.

After reaching the optimal position of the screws in the two different pelvic bone corridors, the angles in relation to the sagittal and axial plane of the pelvis were measured. 

### Statistical Analysis

Statistical analysis was carried out using SPSS software (IBM, Armonk, NY, USA). The independent *t*-test was used to compare continuous variables after determining the distribution was appropriate for parametric testing. In this study, *p*-values < 0.05 were considered significant.

## 3. Results

### 3.1. Screw-TIFIcc

The mean angle of both left and right screw related to the axial plane was 63.4° (±1.8°) and to the sagittal plane was 12.3° (±1.5°).

The mean angle of the left side related to the axial plane was 64.6° (±2.0°) and to the sagittal plane was 12.3° (±1.5°). There was no significant difference to the right side with a mean angle of 63.3° (±1.7°) (*p* = 0.557) related to the axial plane and 13.0° (±1.4°) related to the sagittal plane (*p* = 0.053).

We found a significant difference between male and female patients concerning both angles. The mean angle in female patients related to the axial plane was 64.0° (±1.5°) and to the sagittal plane was 12.1° (±1.5°). Male patients had a mean angle of 62.8° (±2.0°) (*p* = 0.002) related to the axial plane and 13.2° (±1.2) (*p* = 0.000) related to the sagittal plane.

### 3.2. Screw-TIFIdv

The mean angle of both left and right screws related to the axial plane was 16.1° (±1.2°) and to the sagittal plane was 20.1° (±2.0°).

The mean angle of the left side related to the axial plane was 16.0° (±2.0°) and to the sagittal plane was 20.6° (±1.8°). There was no significant difference to the right side, with a mean angle of 16.2° (±1.4°) (*p* = 0.504) related to the axial plane and 21.0° (±2.1°) related to the sagittal plane (*p* = 0.453). 

We found a significant difference between male and female patients concerning both angles. The mean angle in female patients related to the axial plane was 16.5° (±1.3°) and to the sagittal plane was 10.3° (±1.7°). Male patients had a mean angle of 15.7° (±1.1°) (*p* = 0.003) related to the axial plane and 21.3° (±2.1°) (*p* = 0.012) related to the sagittal plane.

Statistical investigation showed that increasing the number of participants would not change the results. 

## 4. Discussion

Anatomical and morphometric analysis based on CT scans as well as performed on human cadaver bodies are important to develop innovative surgical procedures and to guarantee their success. Especially taking into account the unique three-dimensional shape and the complex anatomy of the true pelvis, thorough consideration and careful preparation are required before performing percutaneous surgical techniques.

In numerous publications, diverse bone canals of the pelvis have been described for hosting screws for implant fixation and fracture stabilization [14,15,16,17,18]. In the majority of the publications, the length and the width of these bone corridors were analyzed.

Schildhauer described a pelvic bone corridor above the acetabulum with a length of 140 mm and a width of 8 mm [12]. Schmitz et al. emphasized that this corridor leading from the PIIS to the AIIS can be used to host Schanz screws to perform a cement augmented transiliac internal fixation in order to treat instable posterior pelvic ring fractures in geriatric patients [10]. 

To our knowledge, this study is the first investigation analyzing the angles of the supraacetabular bone corridor in relation to the anterior pubic plane. The APP was established and investigated to perform computer-assisted implant navigation in orthopedic surgery [13,19]. It can easily be identified in a prone position [20]. Furthermore, it provides an accurate and reproducible referencing plane in 3D analyses [13].

The knowledge of the orientation of a pelvic bone corridor to host an iliac screw in relation to the APP gives the pelvic trauma surgeon a guide to place the ilium screws of the minimally invasive TIFI procedure in a safe manner.

The outstanding finding of this investigation is that there is hardly any inter-individual diversity. Obviously, the morphological shape of the pelvic ring irrespective of the size of the pelvis is configured in a manner that the supraacetabular corridor spreads out in regular consistent angulation to the axial and the sagittal plane. Even though we did not measure the size of the different pelvises, it has to be assumed that by randomization the relevance for the inter-individual diversity in size and height for each gender group was excluded. The high constancy of the supraacetabular bone canal angulation is unique in contrast to other bone corridors that are used to host screws for posterior pelvic ring osteosynthesis. The most common technique worldwide to stabilize posterior pelvic ring fractures is the SI-screw [6,16]. Anatomical shape variation of the upper sacrum—so-called dysplastic pelvises—occur at a considerable rate of 35% to 54% [21,22]. This frequently leads to screw malposition or even prevents screw placement in S1 [14,23]. Even though we did not analyze the anatomical sacral shape, it has to be assumed that by number and by randomization dysmorphic pelves are included in our analyzed cohort, implicating that a dysmorphic sacrum does not influence the position of the supraacetabular bone corridor.

Apart from the outstanding finding that the supraacetabular bone corridor shows a significant inter-individual constancy, our data revealed a significant difference between male and female pelvises. This is in accordance with other studies providing evidence that gender is a potential source of variability among individuals [20,24,25]. 

### Limitations

Certainly, several limitations of the resent study have to be outlined. First of all, this study is an experimental investigation based on CT scans, which allows for a higher precision within the results than manual measurements in cadaveric or clinical investigations. Nevertheless, the high precision of a tenth of a degree as measured certainly will not be of clinical relevance.

Furthermore, it is assumed that the reference plane, the APP, is horizontal to the floor when the patient is placed in prone position. Even though the APP can easily be identified [20], Sendtner et al. revealed that its clinical reproducibility in a lateral decubitus position led to inaccuracy of the implant position [19].

Last but not least, the resent investigation was performed with intact pelves. In case of a fractured sacrum or a disrupted sacroiliac joint, the potential displacement of the iliac bone consequently changes the course of the supraacetabular bone canal. However, a major indication for the TIFIdv is the insufficiency fracture of an osteoporotic pelvic ring [10], which hardly ever leads to a significant dislocation [11].

## 5. Conclusions

The present investigation shows that bone corridors in the iliac wing spread out in regular angulation to the axial and the sagittal plane irrespective of age and individual morphologic characteristics but varies according to gender. Due to its high inter-individual constancy in length, width [12,26], and angulation these bone corridors are reliable to host screws for posterior pelvic ring fixation irrespective of each individual patient’s pelvic anatomy.

## Data Availability

No new data were created or analyzed in this study. Data sharing is not applicable to this article.

## References

[1] Letournel E. (1993). The treatment of acetabular fractures through the ilioinguinal approach. Clin. Orthop. Relat. Res..

[2] Matta J.M., Tornetta P. (1996). Internal Fixation of Unstable Pelvic Ring Injuries. Clin. Orthop. Relat. Res..

[3] Gänsslen A., Grechenig S., Nerlich M., Müller M. (2016). Standard Approaches to the Acetabulum Part 1: Kocher-Langenbeck Approach. Acta Chir. Orthop. Traumatol. Cechoslov..

[4] Bastian J.D., Ansorge A., Tomagra S., Siebenrock K.A., Benneker L.M., Büchler L., Keel M.J.B. (2015). Anterior fixation of unstable pelvic ring fractures using the modified Stoppa approach: Mid-term results are independent on patients’ age. Eur. J. Trauma Emerg. Surg..

[5] Keel M.J.B., Benneker L.M., Siebenrock K.A., Bastian J.D. (2011). Less Invasive Lumbopelvic Stabilization of Posterior Pelvic Ring Instability: Technique and Preliminary Results. J. Trauma: Inj. Infect. Crit. Care.

[6] Tjardes T., Paffrath T., Baethis H., Shafizadeh S., Steinhausen E., Steinbuechel T., Rixen D., Bouillon B. (2008). Computer Assisted Percutaneous Placement of Augmented Iliosacral Screws. Spine.

[7] Routt M.L.C., Simonian P.T., Grujic L. (1995). Preliminary Report: The Retrograde Medullary Superior Pubic Ramus Screw for the Treatment of Anterior Pelvic Ring Disruptions: A New Technique. J. Orthop. Trauma.

[8] Gänsslen A., Krettek C. (2006). Die retrograde transpubische Schraubenfixation von oberen Schambeinastbrüchen. Oper. Orthopädie Traumatol..

[9] Füchtmeier B., Maghsudi M., Neumann C., Hente R., Roll C., Nerlich M. (2004). The minimally invasive stabilization of the dorsal pelvic ring with the transiliacal internal fixator (TIFI)--surgical technique and first clinical findings. Der Unf..

[10] Schmitz P., Baumann F., Grechenig S., Gaensslen A., Nerlich M., Müller M.B. (2015). The cement-augmented transiliacal internal fixator (caTIFI): An innovative surgical technique for stabilization of fragility fractures of the pelvis. Injury.

[11] Rommens P.M., Hofmann A. (2013). Comprehensive classification of fragility fractures of the pelvic ring: Recommendations for surgical treatment. Injury.

[12] Schildhauer T.A., McCulloch P., Chapman J.R., Mann F.A. (2002). Anatomic and Radiographic Considerations for Placement of Transiliac Screws in Lumbopelvic Fixations. J. Spinal Disord. Tech..

[13] Lass R., Kubista B., Olischar B., Frantal S., Windhager R., Giurea A. (2014). Total Hip Arthroplasty Using Imageless Computer-Assisted Hip Navigation. J. Arthroplast..

[14] Gardner M.J., Morshed S., E Nork S., Ricci W.M., Routt M.L.C. (2010). Quantification of the Upper and Second Sacral Segment Safe Zones in Normal and Dysmorphic Sacra. J. Orthop. Trauma.

[15] Moshirfar A., Rand F.F., Sponseller P.D., Parazin S.J., Khanna A.J., Kebaish K.M., Stinson J.T., Riley L.H. (2005). Pelvic Fixation in Spine Surgery. J. Bone Jt. Surg. -Am. Vol..

[16] Nork S.E., Jones C.B., Harding S.P., Mirza S.K., Routt M.L.C. (2001). Percutaneous Stabilization of U-Shaped Sacral Fractures Using Iliosacral Screws: Technique and Early Results. J. Orthop. Trauma.

[17] Mehling I., Hessmann M.H., Rommens P.M. (2012). Stabilization of fatigue fractures of the dorsal pelvis with a trans-sacral bar. Operative technique and outcome. Injury.

[18] Vanderschot P., Kuppers M., Sermon A., Lateur L. (2009). Trans-iliac-sacral-iliac-bar procedure to treat insufficiency fractures of the sacrum. Indian J. Orthop..

[19] Sendtner E., Schuster T., Wörner M., Kalteis T., Grifka J., Renkawitz T. (2010). Accuracy of acetabular cup placement in computer-assisted, minimally-invasive THR in a lateral decubitus position. Int. Orthop..

[20] Baumann F., Schmitz P., Mahr D., Kerschbaum M., Gänsslen A., Nerlich M., Worlicek M. (2018). A guideline for placement of an infra-acetabular screw based on anatomic landmarks via an intra-pelvic approach. J. Orthop. Surg. Res..

[21] Chip M.L.C., Simonian P.T., Agnew S.G., Mann F.A. (1996). Radiographic Recognition of the Sacral Alar Slope for Optimal Placement of Iliosacral Screws: A Cadaveric and Clinical Study. J. Orthop. Trauma.

[22] Kim J.-J., Jung C.-Y., Eastman J.G., Oh H.-K. (2016). Measurement of Optimal Insertion Angle for Iliosacral Screw Fixation Using Three-Dimensional Computed Tomography Scans. Clin. Orthop. Surg..

[23] Mendel T., Radetzki F., Wohlrab D., Stock K., Hofmann G.O., Noser H. (2013). CT-based 3-D visualisation of secure bone corridors and optimal trajectories for sacroiliac screws. Injury.

[24] Handa V.L., Lockhart M.E., Fielding J.R., Bradley C.S., Brubakery L., Cundiffy G.W., Ye W., Richter H.E. (2008). Racial Differences in Pelvic Anatomy by Magnetic Resonance Imaging. Obstet. Gynecol..

[25] Gras F., Gottschling H., Schröder M., Marintschev I., Reimers N., Burgkart R. (2015). Sex-specific Differences of the Infraacetabular Corridor: A Biomorphometric CT-based Analysis on a Database of 523 Pelves. Clin. Orthop. Relat. Res..

[26] Miller F., Moseley C., Koreska J. (1990). Pelvic anatomy relative to lumbosacral instrumentation. J. Spinal Disord..

